# LINC00470 accelerates the proliferation and metastasis of melanoma through promoting APEX1 expression

**DOI:** 10.1038/s41419-021-03612-z

**Published:** 2021-04-19

**Authors:** Ting Huang, Yong-Jie Wang, Mi-Tao Huang, Yu Guo, Li-Chang Yang, Xiao-Jin Liu, Wu-Yuan Tan, Jian-Hong Long

**Affiliations:** 1 Department of Plastic Surgery, Xiangya Hospital, Central South University, Changsha, 410011 Hunan Province P. R. China; 2 Department of Burns and Reconstructive Surgery, Xiangya Hospital, Central South University, Changsha, 410011 Hunan Province P. R. China

**Keywords:** Cell biology, Diseases

## Abstract

Recently studies found that APEX1 was abnormally expressed in melanoma, indicating that it might be involved in the development of melanoma. However, the underlying mechanism and the interaction between APEX1 and LINC00470 in melanoma are not clear. Therefore, we aimed to investigate the role of LINC00470 in the development of melanoma in this work. We discovered that LINC00470 was overexpressed in melanoma tissues and cells compared with the adjacent normal tissues and cells by qPCR. The overexpression of LINC00470 promoted the proliferation and migration of melanoma cells. The functional investigation demonstrated that LINC00470 activated the transcription factor, ZNF131, to regulate the APEX1 expression, which finally promoted cell proliferation and migration. In contrast, knockdown of LINC00470 could significantly inhibit the melanoma cell proliferation and migration, and suppress the growth of tumor in vivo. Overexpression of APEX1 could reverse the impact of the silence of LINC00470 in melanoma cells. In summary, our studies revealed that LINC00470 promoted melanoma proliferation and migration by enhancing the expression of APEX1, which indicated that LINC00470 might be a therapeutic target for the treatment of melanoma.

## Introduction

Melanoma is recognized as the most aggressive skin cancer with rapid progression, stemming from melanocytes^1,2^. Every year, the incidence of melanoma is rapidly increased^3^. Around the world, melanoma ranks as the seventh most common cancer for females and the fifth most common cancer in male^4^. Melanoma is also characterized by the most common cancer with metastasis. Patients are usually diagnosed with melanoma at a late stage with metastasis, resulting in a poor prognosis. The 5-year survival rate of melanoma was reported to be less than 18%^5,6^. Therefore, it is urgent and important to explorer the molecular mechanism of the development of melanoma, which might provide potential therapeutic targets for melanoma treatment.

Apurinic/apyrimidinic endonuclease 1/redox effector factor 1 (APEX1), an important multifunctional protein, not only works as a transcriptional coactivator in cell apoptosis, proliferation, and differentiation but also regulates DNA repair and adjustment of intracellular reactive oxygen species^7,8^. Therefore, APEX1 has been studied and worked as a biomarker for a variety of cancers. For example, Cao et al.^9^ found that APEX1 was overexpressed in the hepatocellular carcinoma (HCC) tissues and cells, which indicated that APEX1 could be used for the diagnosis and prognosis of HCC. APEX1 was also upregulated in non‑small‑cell lung cancer and co-related with the high cell proliferation and metastasis^10^. Besides these two types of cancer, abnormal expression of APEX1 in both mRNA and protein levels have been found in other cancers, such as breast cancer^11^, gastric cancer^12^, ovarian serous cancer^13^, bladder cancer^14^, colon cancer^15^, prostate cancer^16^, osteosarcoma cancer^17^, melanoma^18^, etc. Overexpression (OE) of APEX1 in melanoma was clearly observed by both Yang et al.^18^ as well as Figi et al.^19^, showing that APEX1 might involve in the development of melanoma. However, the regulation of APEX1 in melanoma and its mechanism is unclear, which limited the discovery of its related potential therapeutic strategies.

Long non-coding RNAs (LncRNAs) with no protein-coding ability, are a new type of RNAs with more than 200 nucleotides. LncRNAs act as functional regulators in a variety of cancers^20,21^. LncRNAs regulate target gene expression and conduct their function in cancer through various mechanisms, such as chromatin modification, RNA editing/splicing/degradation, miRNA sequestration, transcriptional activation/repression, and translational efficiency modulation^22^. In melanoma, LncRNAs also play important roles in cell proliferation, invasion, and migration. For example, Chen et al.^23^ found that LncRNA GAS5 could inhibit tumor growth through regulating the metastasis of melanoma cells. Zhang et al.^6^ discovered that LncRNA HOXD-AS1 promoted melanoma cell proliferation and invasion by inhibiting the expression of RUNX3. LINC00470, identified as C18orf2, is a LncRNA located in chromosome band 18p11.32^24,25^. Recently, LINC00470 was found highly expressed in the glioblastoma tissues and cells, which activated AKT signaling and promoted the expression of ELFN2 to inhibit cell autophagy and induce tumorigenesis^26,27^. Yan et al. found that LINC00470 was significantly upregulated in gastric cancer cells compared with normal tissues and cells. Meanwhile, they demonstrated that LINC00470 induced the degradation of PTEM mRNA to promote gastric cancer proliferation, migration, and invasion^28^. However, the functions and molecular mechanisms of LINC00470 remain unclear in melanoma.

In the study, we found that LINC00470 and APEX1 were significantly overexpressed in the tissues and cells of melanoma. Furthermore, upregulation of LINC00470 significantly promoted cell proliferation and migration. Knockdown of LINC00470 in vivo reduced tumor growth. In addition, we discovered that LINC00470 activated the transcription factor ZNF131 to promote the OE of APEX1 in protein level, but not in mRNA level. Therefore, we believe that LINC00470 may be used as a potential therapeutic target of melanoma.

## Materials and methods

### Tissue specimens and cell lines

Melanoma cancer tissues and healthy tissues from 20 patients were obtained during tumor removal surgery with their signed statement of informed consent. Those tissues were kept at −80 °C in liquid nitrogen before usage. Approvements were made by the ethics committee of Central South University.

The human melanoma cell lines, including A375, A875, SK-MEL-1, SK-MEL-5, and SK-MEL-28, as well as normal human epidermal melanocytes (PIG1), were obtained from the American Type Culture Collection (ATCC, Manassas, VA). RPMI-1640 or DMEM or 254 medium supplemented with 10% fetal bovine serum (FBS) were used to culture cells at 37 °C in a humidified chamber with 5% CO_2_.

### Cell transfection

All the short hairpin (sh) RNAs, including shNC, sh-APEX1, shLINC00470, and shZNF131 were synthesized by GenePharma (Shanghai, China). The following shows all the sequences: shNC: 5′-UCUAUGUGUCUUAAUCCCUUGUCCU-3′; sh-APEX1: 5′-GACACGCGACUUGUACCAC-3′; shLINC00470: 5′-GACACGCGACUUGUACCAC-3′; shZNF131: 5′-GACACGCGACUUGUACCAC-3′. In addition, pcDNA3.1 empty vector (Invitrogen, Shanghai, China) was inserted with the cDNA sequences of AR, APEX1, LINC00470, and ZNF131 to construct the OE vectors, including OE-AR, OE-APEX1, OE-LINC00470, and OE-ZNF131, respectively. Lipofectamine 3000 transfection reagent (Invitrogen) was used to transfect cells following the manufacturer’s instructions.

### Quantitative real-time polymerase chain reaction (qRT-PCR)

TRIzol reagent (Invitrogen, Carlsbad, CA) was used to collect total RNA. Then, the PrimeScript^TM^ RT Reagent kit (Takara Bio. Inc) was utilized to obtain the cDNA through reverse transcription. All the primers were obtained from GenePharma (Shanghai, China). The DNA primers sequences: APEX1 forward, 5′-tgggcttccagagctttcta-3′ and reverse, 5′-ttctttattgagggcaaccg-3′; LINC00470 forward, 5′-agggacaatctcacacagg-3′ and reverse, 5′-gactcaaccttcctctcca-3′; ZNF131 forward, 5′-agggacaatctcacacagg-3′ and reverse, 5′-gactcaaccttcctctcca-3′; GAPDH forward, 5′-gtttgtgatgggtgtgaacc-3′ and reverse, 5′-tcttctgagtggcagtgatg-3′. mRNA levels were determined by qRT-PCR on an ABI Prism 7900HT (Applied Biosystems, Foster City, CA USA). The ΔΔCq method was used to quantify the Cq-value for every sample, whose results were expressed as 2^−ΔΔCq^. Each sample was performed in triplicate.

### LncRNA sequencing

LncRNA sequencing was applied to analyze the expression of LncRNAs in the AR-overexpressed A375 cells and A375 cells. First, total RNAs were collected by TRIzol reagent (Invitrogen). Ribosomal RNA (rRNA) was discarded using Epicenter Ribo-Zero^TM^ rRNA Removal Kit (Epicenter, USA) from the total RNA. Then, cDNA libraries were obtained with the Illumina TruSeq Stranded Total RNA using RiboZero Human Kit (Illumina, San Diego, CA). Finally, the sequencing was performed by Illumina HiSeq. 2000 platform. The RNA-seq dataset was analyzed and demonstrated by the Integrative Genomics Viewer.

### RNA immunoprecipitation (RIP) assay

RIP assay was utilized to investigate the relationship between LINC00470 and transcription factor ZNF131 in melanoma tissues and cells. An EZ-Magna RIP^™^ RNA-Binding Protein Immunoprecipitation Kit (Millipore, USA) and anti-ZNF131 antibody (Invitrogen, PA5-43065 were utilized in the RIP assay following the manufacturer’s instructions. Melanoma tissues and cells were lysed by the buffer provided in the kit. Then, anti-ZNF131 or control IgG (negative control) was applied for immunoprecipitation. qRT-PCR was conducted to quantify the LINC00470 level after purification with RNAiso Plus (Takara, Japan).

### Western blot analysis

Briefly, the cells or tissues were lysed using RIPA lysis buffer (Beijing Solarbio Science & Technology Co., Ltd., Beijing, China). The total proteins were quantified with a BCA assay kit (Pierce; Fisher Scientific, Inc.). The antibodies were used as follows: anti-APEX1 (1:10,000, Proteintech, 10203-1-AP, anti-ZNF131 (1:10,000, Invitrogen, PA5-43065), anti-AR (1:10,000, Abcam) and anti-GAPDH (1:10,000; cat. no. ab181602; Abcam). At room temperature, the membranes were incubated with all the primary antibodies for 2 h. Then, PBS was used to wash the membrane three times, followed by incubating with secondary antibodies for another 1 h at room temperature. The signals were obtained through the Chemiluminescence Western blot system (Pierce, Biotechnology Inc., Rockford, USA). Each sample repeated at least three times.

### MTT assay

Cell proliferation was assessed by MTT assay using MTT reagent (Roche Molecular Biochemicals, Rotkreuz, Switzerland). Totally, 2000 cells were seeded in 96-well plates per well for different treatments. After different treatments for 24, 48, and 72 h, the MTT reagent was pipetted into each well and incubated for another 4 h. After removing the medium, 200 µL DMSO was injected into each well. The absorbance at 490 nm was collected on a microplate reader.

### Colony formation assay

The cells with a density of 5000 cells/well were seeded in a 6-well plate for different treatments for 15 days. Then, methanol was used to fix the cells, followed by the incubation of 0.1% crystal violet. The colonies were observed and quantified with the optical microscope. Each sample was repeated in triplicates.

### Transwell assay

A Transwell assay was utilized to determine the migration ability of cells with a two-chamber with an 8 µm pore size membrane filter (Corning, Cambridge, MA). Briefly, cells with different treatments were seeded in the top chamber in the serum-free medium. In the lower chamber, a complete medium containing 10% FBS was used. We incubated the chambers at 37 °C for 48 h. After removing the cells in the top chamber, the cells in the lower chamber were fixed with methanol, after which 0.1% crystal violet was used to stain the cells for 30 min. Then, the stained cells were imaged and counted under the microscope. Three independent experiments were conducted for each sample.

### Luciferase reporter assay

The luciferase reporter assay was conducted according to the previous literature^27^. The cells were co-transfected with psiCHECK2 plasmid (Promega, Madison, WI) containing the APEX1 promoter and ZNF131 wild-type (WT) or mutant for 48 h. Dual-Glo Luciferase Assay System (Promega) was used to quantify the luciferase reporter activity following the manufacturer’s protocol.

### Generation of xenografts

The animal experiments were approved by the Animal Ethics Committee of Central South University. Briefly, xenografts were generated by injecting the A375 cells (2 × 10^6^ in 0.1 ml PBS) subcutaneously into six-week-old BALB/c male athymic nude mice (*n* = 5/group, Vitalriver, Beijing, China). Then, the tumor sizes were monitored for 28 days every 7 days (tumor volume = (length × width^2^)/2). On day 28, the tumor tissues from each group were collected after the mice were sacrificed. The obtained tissues were used for further analysis.

### IHC analysis

The resected tumor tissues from each group were fixed using 10% formalin and stored in paraffin for section. Then, the samples were stained with hematoxylin and eosin. The anti-ZNF131 antibody (diluted at 1:100, Invitrogen, PA5-43065), anti-Ki67 antibody (diluted at 1:500, Abcam, ab15580) and anti-APEX1 antibody (diluted at 1:100, Proteintech, 10203-1-AP) were utilized for IHC staining according to the manufacture’s instruction.

### RNA pull-down

RNA pull-down assay was also used to investigate the relationship between LINC00470 and transcription factor ZNF131 in melanoma cells. The biotin-coupled RNA complex was pulled down by incubating the melanoma cell lysates with streptavidin-coated magnetic beads (Sigma, USA) following the manufacturer’s instructions. Real-time PCR analysis was used to evaluate the enrichment of LINC00470 in the capture fractions. LINC0047 junction probe, control probe is ordered from Sangon Biotech (Shanghai, China). The proteins in the capture complex were identified by western blotting using an anti-ZNF131 antibody.

### Chromatin Immunoprecipitation (CHIP) assay

To study the relationship between transcription factor ZNF131 and APEX1 in melanoma cells, we performed a CHIP assay using a SimpleChIP^®^ Enzymatic Chromatin IP Kit (CST, USA). Briefly, the cultured melanoma cells were lysed and sonicated to chromatins. The chromatins were incubated with anti-ZNF131 antibodies or normal rabbit IgG overnight. Real-time PCR analysis was used to quantify the APEX1 level in the enrichment of specific DNA sequences. The primers used for ChIP are ordered from Sangon Biotech (Shanghai, China).

### Statistical analysis

Data are shown as the means ± standard deviation (SD). Every group was repeated at least three times. Student’s *t* test or one-way ANOVA was utilized to compare the difference. *p* < 0.05 was defined as statistically significant differences (*p < 0.05, **p < 0.01, and ***p < 0.001).

## Results

### APEX1 promoted the melanoma cell proliferation and migration

**Fig. 1:**
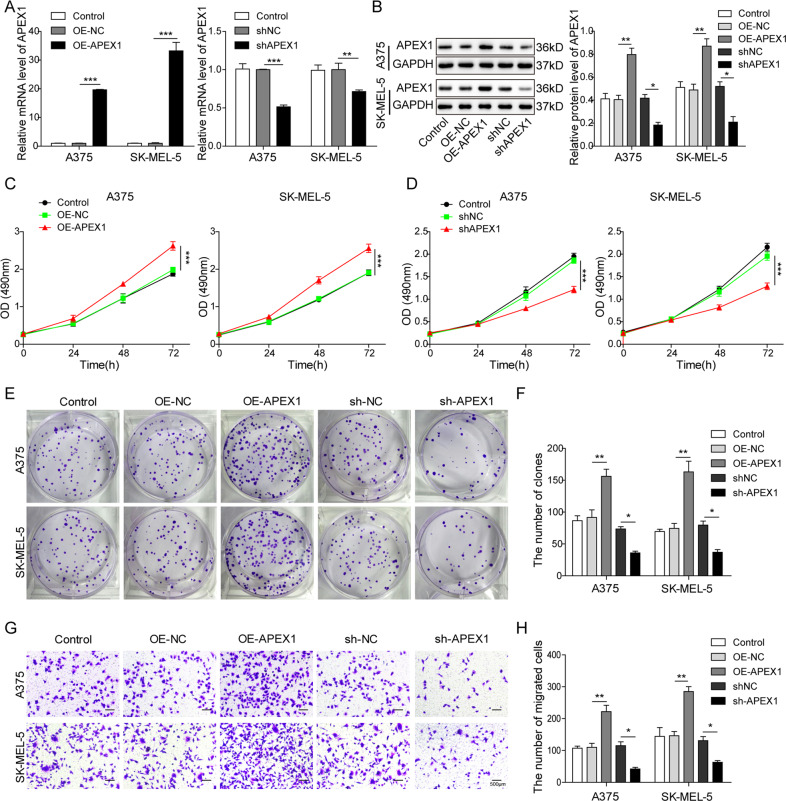
APEX1 facilitated the proliferation and migration of melanoma cells. **A**, **B** qRT-PCR and WB analysis of APEX1 of A375 and SK-MEL-5 cells after transfection with OE-NC, OE-APEX1, shNC, and sh-APEX1, respectively. **C** MTT assay of the cells transfected with control, OE-NC, and OE-APEX1. **D** MTT assay of the cells transfected with control, sh-NC, and sh-APEX1. **E**, **F** The colony formation assay of A375 and SK-MEL-5 cells after transfected with control, OE-NC, OE-APEX1, sh-NC, and sh-APEX1. **G**, **H** The transwell assay was used to evaluate the migration ability of the cells after transfected with control, OE-NC, OE-APEX1, sh-NC, and sh-APEX1. The mean ± SD in the graph shows the relative levels from three replicates. *p < 0.05, **p < 0.01.

To investigate the function of APEX1 in melanoma, we transfected the OE or silence vector of APEX1 into the A375 and SK-MEL-5 cells. As shown in Fig. 1A, B, A375 and SK-MEL-5 cells transfected with OE-APEX1 showed significantly enhanced expression of APEX1 compared with the OE-NC group. In contrast, transfection with sh-APEX1 significantly decreased the expression of APEX1 compared with shNC group. These results indicated that the OE or silence of APEX1 was successfully fulfilled in A375 and SK-MEL-5 cells. MTT and colony formation assay was used to assess the cell proliferation after overexpressing or silencing APEX1. The results demonstrated that cell proliferation was promoted when the APEX1 was overexpressed in A375 and SK-MEL-5 cells (Fig. 1C–F). In contrast, silencing APEX1 significantly suppressed cell proliferation (Fig. 1C–F). Furthermore, the impact of APEX1 on migration ability was investigated using transwell assay in the melanoma cells. We found that the OE of APEX1 significantly promoted the cell migration ability (Fig. 1G, H). Inversely, the knockdown of APEX1 suppressed cell migration of A375 and SK-MEL-5 cells (Fig. 1G, H). Overall, these results indicated that APEX1 promoted the proliferation and migration ability of melanoma cells.

### Linc00470 overexpressed in the melanoma tissues and cells

**Fig. 2:**
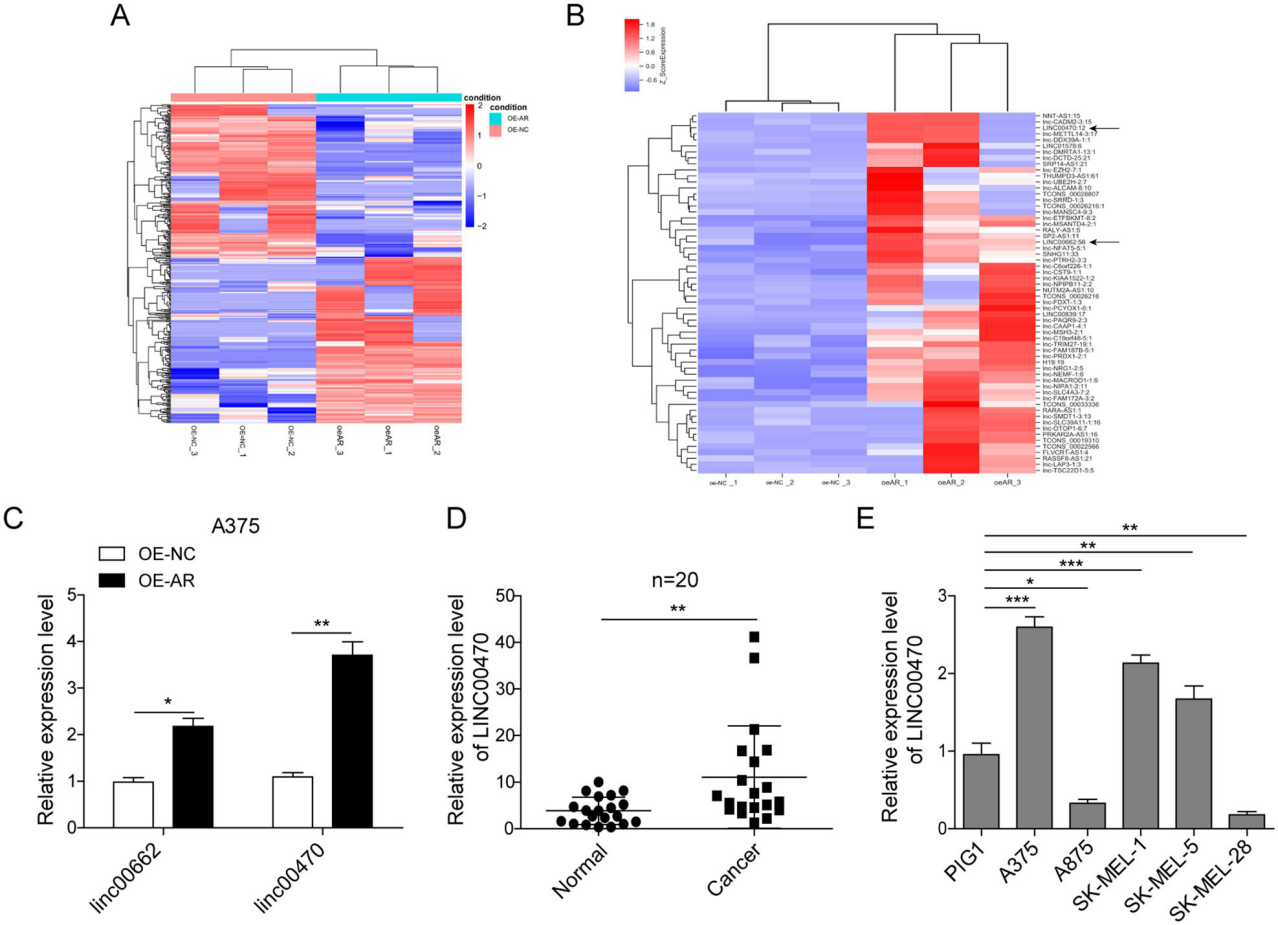
LINC00470 was upregulated in the AR-overexpressed A375 cells. **A**, **B** Heatmap generated by LncRNA sequencing from A375 cells and AR-overexpressed A375 cells. **C** The expression of LINC00662, and LINC00470 were analyzed in the A375 cells and AR-overexpressed A375 cells using qRT-PCR. **D** Expression of LINC00470 in the normal and melanoma tissues (*n* = 20) analyzed by qRT-PCR. **E** The expression of LINC00470 in different cell lines was quantified by qRT-PCR. The mean ± SD in the graph shows the relative levels from three replicates. *p < 0.05, **p < 0.01.

Androgen receptor (AR) involves in the development of melanoma. To discover the function of the lncRNAs in the AR-induced melanoma development, we studied the expression of lncRNAs in AR-overexpressed melanoma cells using lncRNA sequencing analysis. Many abnormally expressed lncRNAs in the AR-overexpressed A375 cells were identified (Fig. 2A). LINC00662 and LINC00470 ranked as one of the top 60 remarkably upregulated expressed lncRNAs with relatively high abundance (Fig. 2B), which was validated by qRT-PCR analysis (Fig. 2C). Meanwhile, we detected LINC00470 expression in 20 paired melanoma tissue and adjacent normal tissue by qRT-PCR, and the expression of LINC00470 was significantly higher in melanoma tissues (Fig. 2D). In addition, LINC00470 expression was upregulated in three melanoma cells (A375, SK-MEL-1, and SK-MEL-5), compared with the human normal skin melanocytes cell line PIG1 (Fig. 2E). Taken together, these results demonstrated that the LINC00470 was upregulated in melanoma tissues and cells, which might be used as a melanoma prognostic marker.

### LINC00470 promoted the proliferation and migration of melanoma cells

**Fig. 3:**
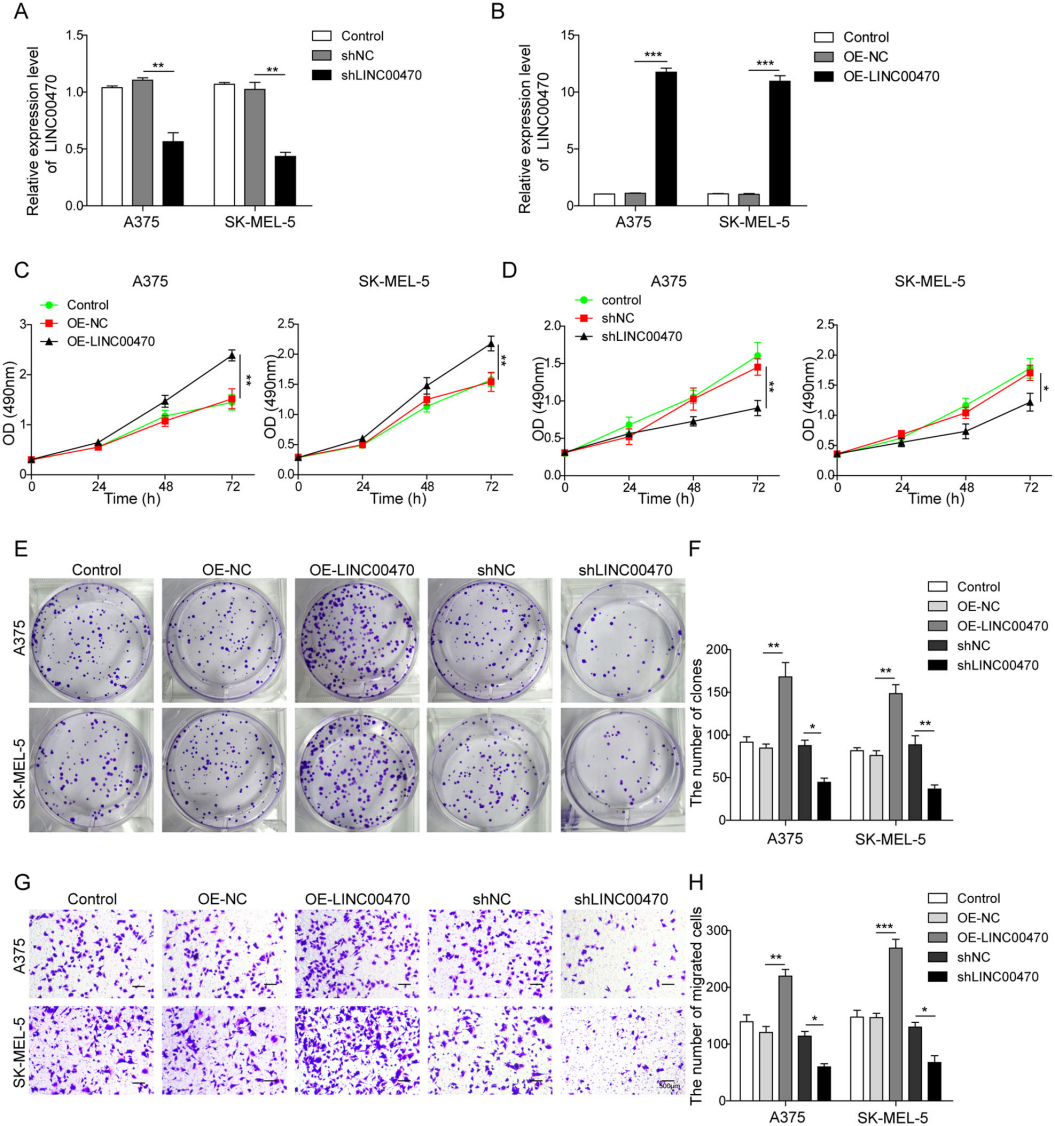
LINC00470 facilitated the proliferation and migration of melanoma cells. **A**, **B** qRT-PCR analysis of LINC00470 of cells transfected with shNC, shLINC00470, OE-NC, and OE-LINC00470, respectively. **C** MTT assay of the cells after transfected with OE-NC and OE-LINC00470, respectively. **D** MTT assay of the after transfected with shNC and shLINC00470, respectively. **E**, **F** The colony formation assay of the cells after transfected with control, OE-NC, OE-LINC00470, sh-NC, and shLINC00470. **G**, **H** Migration ability of the cells after transfected with control, OE-NC, OE-LINC00470, sh-NC, and shLINC00470 evaluated by transwell assay. The mean ± SD in the graph shows the relative levels from three replicates. *p < 0.05, **p < 0.01.

The role of LINC00470 on cell proliferation and migration ability was investigated by overexpressing or silencing LINC00470 in A375 and SK-MEL-5 cells. The qRT-PCR analysis demonstrated that LINC00470 was successfully silenced and overexpressed in A375 and SK-MEL-5 cells, which were transfected with shLINC00470 and OE-LINC00470, respectively (Fig. 3A, B). MTT and colony-forming assay revealed that OE of LINC00470 could significantly promote cell proliferation. In contrast, knockdown of LINC00470 led to significant inhibition of cell proliferation (Fig. 3C–F). Moreover, overexpressing LINC00470 significantly increased melanoma cell migration (Fig. 3G, H). However, knocking down LINC00470 suppressed cell migration ability in A375 and SK-MEL-5 cells (Fig. 3G, H). Therefore, we concluded that LINC00470 promoted cell proliferation and migration in A375 and SK-MEL-5 cells.

### LINC00470 promoted melanoma cell proliferation and migration through increasing APEX1

**Fig. 4:**
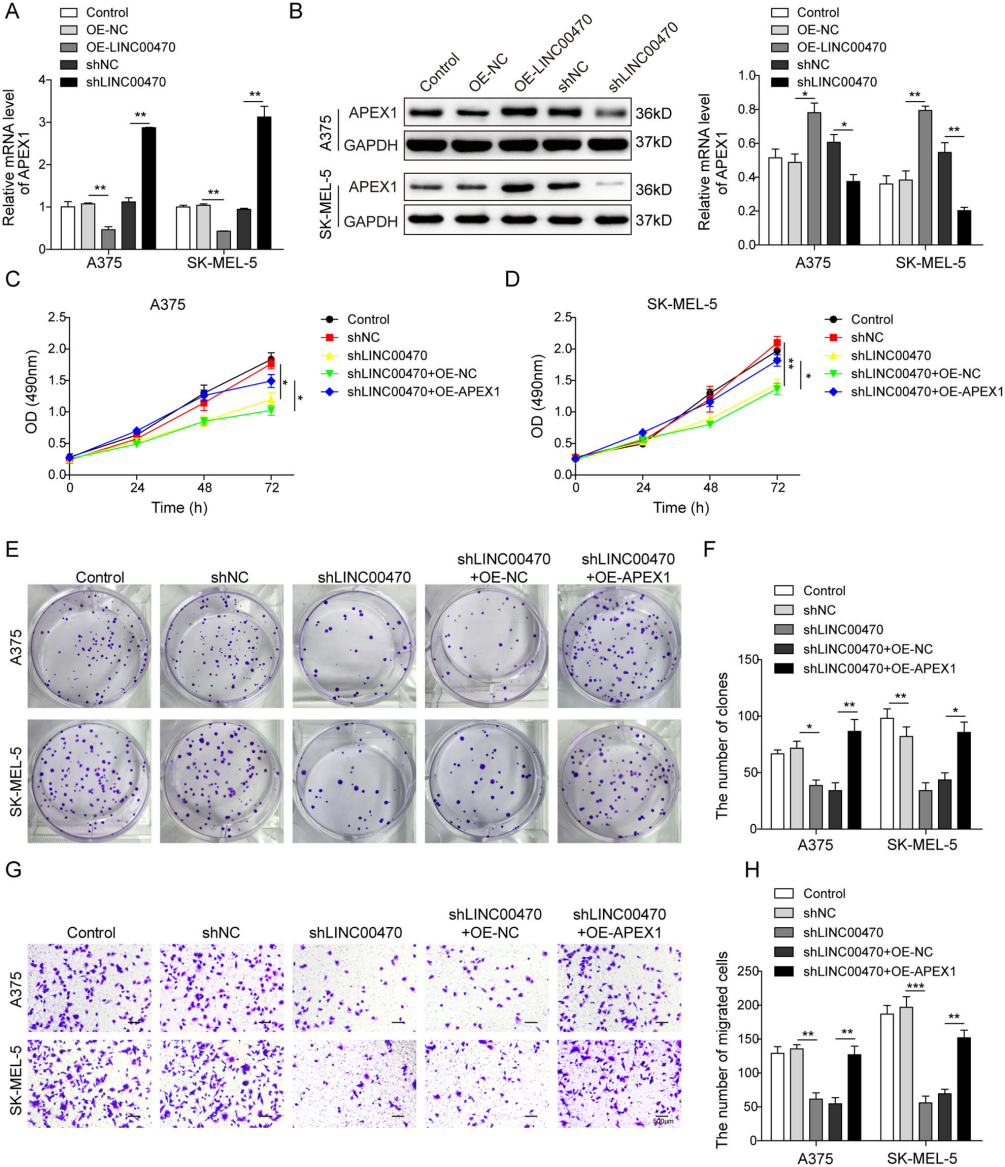
Proliferation and migration of melanoma cells was promoted by LINC00470 through enhancing APEX1. **A** qRT-PCR analysis of APEX1 in cells transfected with OE-NC, OE-LINC00470, shNC, and shLINC00470, respectively. **B** Western blot analysis of APEX1 in cells transfected with OE-NC, OE-LINC00470, shNC, and shLINC00470, respectively. In the following experiments, all the cells were transfected with five different groups, including control, shNC, shLINC00470, shLINC00470 + OE-NC, and shLINC00470 + OE-APEX1, respectively. **C**, **D** The cell proliferation of cells with different treatments analyzed by MTT assay for 24, 48, and 72 h. **E**, **F** The colony formation assay of the cells with different treatments. **G**, **H** Cell migration ability of cells with different treatments analyzed by transwell assay. The mean ± SD in the graph shows the relative levels from three replicates. *p < 0.05*,* **p < 0.01.

From the previous results, we found both LINC00470 and APEX1 were upregulated in melanoma cells. Subsequently, we investigated the detailed relationship between LINC00470 and APEX1 in melanoma cells. In the AR-overexpressed A375 cells, western blot analysis demonstrated that AR and APEX1 were both upregulated (Fig. S1). As shown in Fig. 4A, OE LINC00470 inhibited while knockdown LINC00470 promoted the mRNA expression level of APEX1 in melanoma cells. Interestingly, the protein expression level of APEX1 was increased when the melanoma cells were overexpressed LINC00470 and decreased when LINC00470 was knockdown (Fig. 4B). These results implied that LINC00470 might impact the protein expression level of APEX1 through the regulation of posttranscriptional translation. Then, MTT and colony formation assay demonstrated that silencing LINC00470 suppressed the cell proliferation ability but OE of APEX1 reversed the effect of silencing LINC00470 in cells (Fig. 4C–F). Furthermore, we found that knockdown LINC00470 inhibited the cell migration ability and OE of APEX1 abrogated the impact of silencing LINC00470 in A375 and SK-MEL-5 cells (Fig. 4G, H). All these results demonstrated that LINC00470 promoted cell proliferation and migration partly by enhancing the protein expression of APEX1 in melanoma cells.

### LINC00470 regulated APEX1 expression through activating Zinc finger protein 131 (ZNF131)

**Fig. 5:**
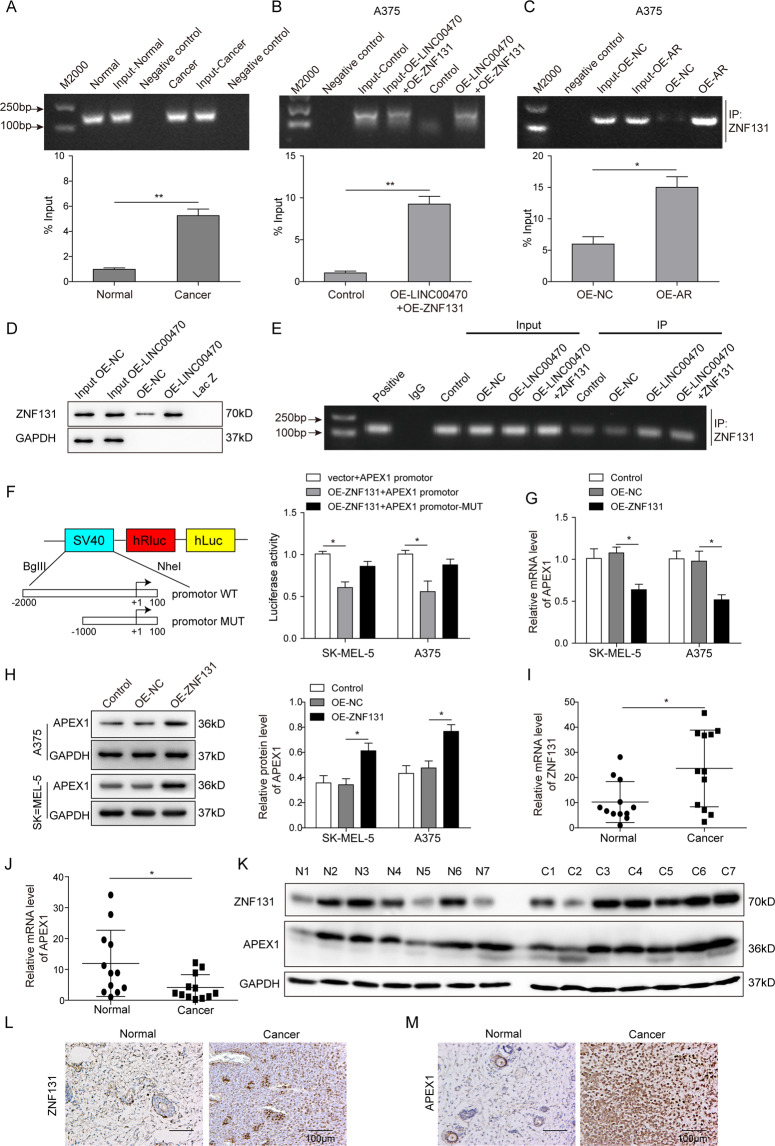
LINC00470 activated ZNF131 to inhibit the transcription of APEX1. **A** The interaction of LINC00470 and ZNF131 was detected by RIP assay in normal and melanoma tissues. **B**, **C** RIP assay showed the enrichment of LINC00470 and ZNF131 in melanoma cells or the AR-overexpressed melanoma cells. **D** RNA pull-down and western blot analysis after the cells were transfected with OE-NC and OE-LINC004705. **E** CHIP assay analysis after the cells were transfected with control, OE-NC, OE-LINC00470, and OE-LINC00470 with OE-ZNF131. **F** Dual-luciferase assay was used to investigate the interaction between ZNF131 and APEX1. **G**, **H** qRT-PCR and WB assay showed the expression level of APEX1 in ZNF131-overexpressed melanoma cells. qRT-PCR analysis of ZNF131 (**I**) and APEX1 (**J**) in melanoma tissues and normal tissues. **K** The protein expression of APEX1 and ZNF131 in melanoma and normal tissues using western blot. IHC assay was conducted to evaluate the levels of ZNF131 (**L**) and APEX1 (**M**) in melanoma and normal tissues. The mean ± SD in the graph shows the relative levels from three replicates. *p < 0.05, **p < 0.01.

To explore the interaction mechanism between LINC00470 and APEX1, we found that the transcription factor binding with LINC00470 might be ZNF131 by bioinformatic analysis. Then, the relationship among LINC00470, APEX1, and ZNF131 in melanoma cells was detailly investigated by RIP assay and dual-luciferase assay. As shown in Fig. 5A, the result verified that LINC00470 were more enriched in the melanoma cancer tissues relative to the normal tissues when enriched by ZNF131 antibody (Fig. 5A). Meanwhile, the enrichment of LINC00470 and ZNF131 was increased when the A375 cells were overexpressed LINC00470 and ZNF131, or AR (Fig. 5B, C). Furthermore, RNA pull-down assay showed that the stronger binding interaction between LINC00470 and ZNF131 was observed when the LINC00470 was upregulated (Fig. 5D). In order to investigate the interaction between ZNF131 and APEX1, the CHIP assay was performed. The result in Fig. 5E demonstrated that there was a strong interaction between ZNF131 and APEX1 when LINC00470 and ZNF131 were overexpressed in A375 cells. Furthermore, a dual luciferase assay showed that ZNF131 OE significantly decreased the luciferase activity of the APEX promotor but not the APEX promotor-MUT (Fig. 5F). In addition, OE ZNF131 suppressed the mRNA expression of APEX1 but promoted the protein levels of APEX1 (Fig. 5G, H). As shown in Fig. 5I, J, the results show that ZNF131 was upregulated while APEX1 was downregulated in melanoma tissues compared with the normal tissue. However, the western blot and IHC analysis demonstrated that both APEX1 and ZNF131 proteins were significantly overexpressed in the melanoma tissues (Fig. 5K–M). Specifically, we noticed that the protein expression of APEX1 was not consistent with mRNA expression level in melanoma cells as same as melanoma tissue. Therefore, we concluded that LINC00470 could activate the transcription factor ZNF131 to suppress the transcription level of APEX1 but promote the expression of APEX1 at the protein level.

### ZNF131 promoted the proliferation and migration of melanoma cells

**Fig. 6:**
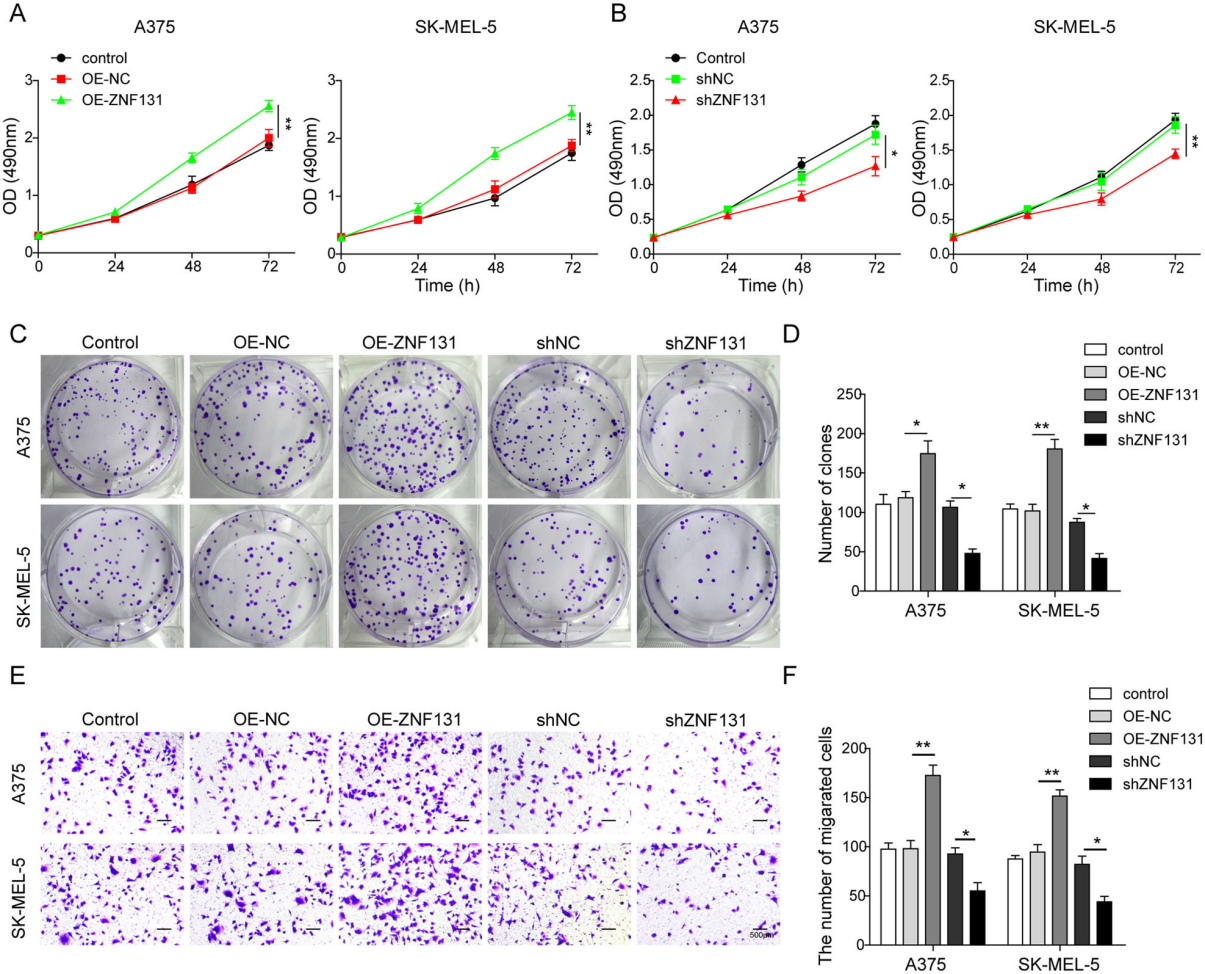
ZNF131 promoted melanoma cell proliferation, migration, and colony formation ability. **A** MTT assay of the cells after transfected with OE-NC and OE-ZNF131, respectively. **B** MTT assay of the cells after transfected with shNC and shZNF131, respectively. **C**, **D** The colony formation assay of the cells after transfected with control, OE-NC, OE-ZNF131, shNC, and shZNF131, respectively. **E**, **F** Migration ability of the cells after transfected with control, OE-NC, OE-ZNF131, shNC, and shZNF131 evaluated by transwell assay. The mean ± SD in the graph shows the relative levels from three replicates. *p < 0.05, **p < 0.01.

To investigate the function of ZNF131 in melanoma, cell proliferation, migration, and colony formation ability were evaluated after the OE or silence of ZNF131 in A375 and SK-MEL-5 cells. As shown in Fig. 6A–D, the OE of ZNF131 significantly promoted the proliferation of melanoma cells. In contrast, cell proliferation was inhibited when ZNF131 was silenced. Furthermore, ZNF131 overexpression improved the cell migration ability while silencing ZNF131 significantly suppressed cell migration (Fig. 6E, F). All these results suggested that the transcription factor ZNF131 could promote melanoma cell proliferation and migration ability.

### LINC00470 promoted melanoma proliferation by enhancing APEX1 expression in vivo

**Fig. 7:**
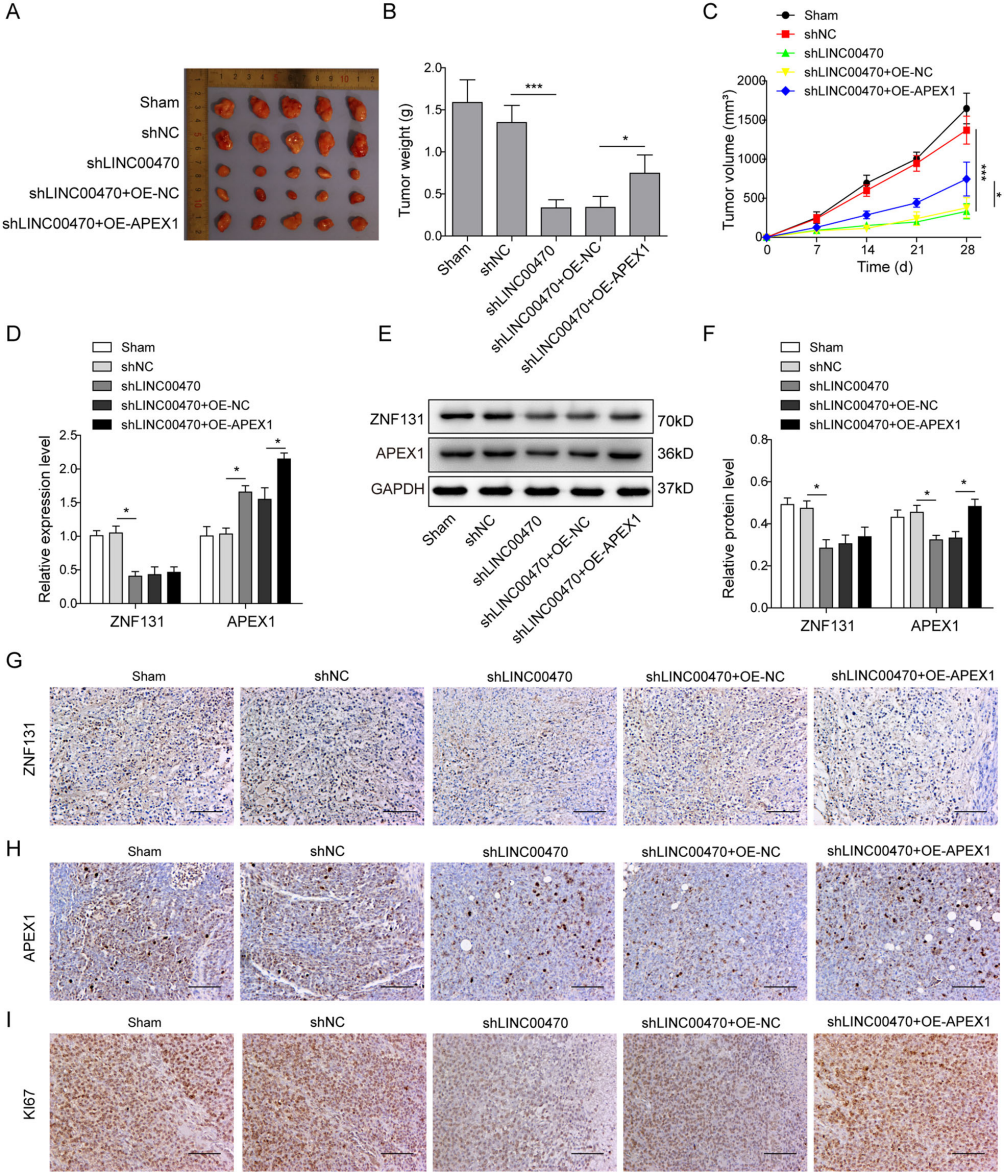
LINC00470 promoted melanoma proliferation and metastasis through enhancing APEX1 expression in vivo. **A** Xenografts were shown after transfecting with shNC, shLINC00470, shLINC00470 + OE-NC, and shLINC00470 + OE-APEX1, respectively. **B** Tumor weight of different groups at day 28. **C** Tumor growth was monitored by analyzing tumor volume for 28 days. **D** Levels of ZNF131 and APEX1 in different groups as described in panel A were analyzed by qRT-PCR. **E**, **F** Levels of ZNF131 and APEX1 in different groups as described in panel (**A**) were analyzed by Western blot. **G**–**I** The immunohistochemical staining was conducted to determine the expression of ZNF131 (**G**), APEX1 (**H**), ki67 (**I**) in different groups as described in panel (**A**). The mean ± SD in the graph shows the relative levels from three replicates. *p < 0.05, **p < 0.01.

All the above results were used to verify the function of LINC00470 in vitro, to investigate the important role of LINC00470 in vivo, A375 cells transfected with shNC, shLINC00470, shLINC00470 with OE-NC, and shLINC00470 with OE-APEX1, were injected into male nude mice subcutaneously to generate the xenograft model in vivo. As shown in Fig. 7A–C, silencing LINC00470 significantly suppressed the growth of the tumor. However, the OE of APEX1 could partly reverse the impact of the silencing LINC00470 (Fig. 7A–C). In addition, we found that knockdown of LINC00470 significantly decreased the mRNA expression of ZNF131 but increased the mRNA expression level of APEX1 (Fig. 7D). However, silencing LINC00470 significantly suppressed the protein expression of ZNF131 and APEX1. Meanwhile, OE of APEX1 significantly enhanced the protein expression of APEX1 but not ZNF131 (Fig. 7E–H). We also found that silencing of LINC00470 significantly suppressed the expression of ki67, which was reversed by OE of APEX1 (Fig. 7I). Overall, these results demonstrated that LINC00470 could promote melanoma tumor growth through regulating APEX1.

## Discussion

In the last decade, LncRNAs have been identified as important regulators in human cancers with different functions^29^. Due to their abnormal expression in tumor tissues and cells, LncRNAs are recognized as a group of promising biomarkers for the diagnosis and prognosis of a variety of cancers^22^. For instance, LncHOTAIR is upregulated in melanoma and is correlated with cancer metastasis and poor prognosis. Knockdown of LncHOTAIR significantly inhibited motility and metastasis^30^. In melanoma tissues and cells, Tian et al.^31^ found that LncRNA MALAT1 was highly expressed. Meanwhile, they demonstrated that the migration ability of melanoma cells was suppressed by silencing LncRNA MALAT1 in vitro. In the current study, we identified that LINC00470 was significantly upregulated in melanoma cells and tissues compared with the normal cells and tissues. The highly expressed LINC00470 was associated with the proliferation and migration ability of advanced melanoma cells, resulting in a rapid tumor growth rate in vivo. Knockdown of LINC00470 significantly suppressed the melanoma cell proliferation and migration, as well as the tumor growth in vivo. Therefore, our results concluded that LINC00470 served as an oncogenic LncRNA in melanoma.

Previously, LINC00470 was found to regulate glioblastoma development through mediating cell autophagy^26,27^. In another report, LINC00470 promoted the HCC proliferation through interaction with NF45/NF90 complex, which regulates gene expression and mRNA stability^25^. In gastric cancer, LINC00470 was also highly expressed and facilitated the malignant behavior of gastric cancer cells through degrading PTEN mRNA^28^. The mechanism of LINC00470 in regulating the proliferation and migration in melanoma is still unknown. Herein, we found that LINC00470 promoted the proliferation and migration of melanoma cells by facilitating the expression of APEX1. APEX1 is a protein that has two functions, including the regulation of DNA repair under oxidative stress and protein reduction–oxidation function^32^. Recently, APEX1 is recognized as a potential diagnostic biomarker in various cancers, such as renal carcinoma, hepatobiliary carcinomas, cholangiocarcinoma, non‑small‑cell lung cancer, etc.^8–10,32^. For example, Wang et al.^10^ showed that the expression of APEX1 was significantly higher in NSCLC tumor tissues than that in the normal tissues, which could be regulated by the miR-296-3p in NSCLC. In the present study, we revealed that LINC00470 was regulating APEX1 to promote the proliferation and migration of melanoma cells, resulting in significant tumor growth.

However, the regulating mechanism of LINC00470 for APEX1 was still unclear in melanoma. Herein, through bioinformatic analysis, RIP assay, and dual-luciferase assay, we identified that LINC00470 activated the transcription factor ZNF131 to regulate APEX1. ZNF131, a member of the zinc finger protein superfamily, is found to act as a transcriptional regulator^33^. In cultured cells, ZNF131 was localized in nuclear, which was found to mediate transcription repression^34^. For instance, Han et al.^35^ reported that ZNF131 inhibited estrogen receptor α (ERα)-mediated transcriptional activity in a dose-dependent manner. Chung et al. demonstrated that ZNF131 bonded with estrogen receptors to suppress estrogen signaling, which further inhibited breast cancer cell proliferation^36^. Ding et al.^37^ discovered that ZNF131 suppressed centrosome fragmentation in glioblastoma by targeting HAUS5. In melanoma cells, we found that ZNF131 targeted APEX1, which finally promoted melanoma cell proliferation and migration. Interestingly, we noticed that the ZNF131 inhibited the mRNA level of APEX1. In contrast, the protein level of APEX1 was upregulated in melanoma tissues, indicating that other regulatory mechanisms to the target gene may exist except the LINC00470/ZNF131/APEX1 axis. The detailed mechanism needs further investigation in the future.

LncRNAs involve in biological processes by a variety of mechanisms, such as chromatin remodeling, cell cycle control, mRNA decay, splicing regulation, and translational regulation^38,39^. However, it is the first time to discover that LINC00470 regulates the target gene through activating the transcription factor, to the best of our knowledge. Therefore, we believe that our present study promotes the understanding of the mechanism of LncRNA in regulating the pathogenesis of melanoma.

In summary, our study demonstrated that LINC00470 could serve as a promising potential therapeutic target for melanoma patients. The detailed investigation about the molecular mechanisms indicated that LINC00470 promoted the proliferation and migration of melanoma cells by facilitating the APEX1 via regulating ZNF131.

## Supplementary information

figure s1

supplementary information
