# Supplementary figures and images for: LINC00470 accelerates the proliferation and metastasis of melanoma through promoting APEX1 expression
Source: Cell Death Dis. 2021 Apr 19;12(5):410. doi: 10.1038/s41419-021-03612-z (PMC8055894; doi:10.1038/s41419-021-03612-z)

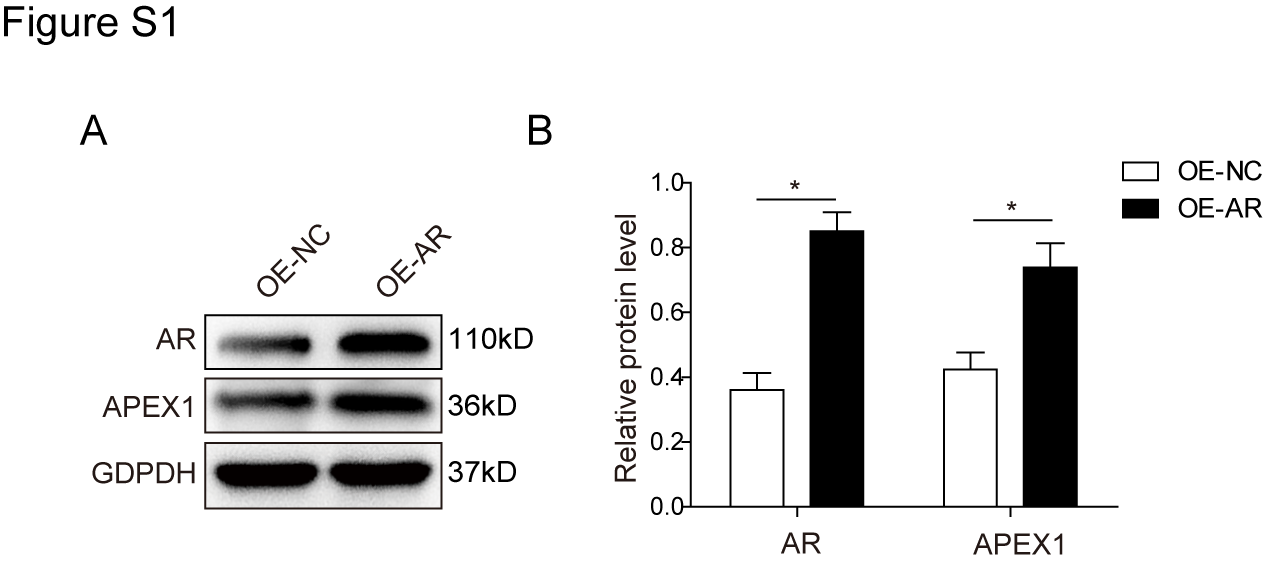

Supplement: Supplementary file 1 — figure s1 [file 41419_2021_3612_MOESM1_ESM.tif]
