# Supplementary material for: LINC00470 accelerates the proliferation and metastasis of melanoma through promoting APEX1 expression
Source: Cell Death Dis. 2021 Apr 19;12(5):410. doi: 10.1038/s41419-021-03612-z (PMC8055894; doi:10.1038/s41419-021-03612-z)
Supplement: Supplementary file 2 — supplementary information [file 41419_2021_3612_MOESM2_ESM.docx]

**Figure S1. ZNF131 and APEX1 was upregulated in AR-overexpressed A375 cells.** (A-B) The expression of AR and APEX1 in AR-overexpressed A375 cells and A375 cells were analyzed by Western blot. GAPDH was used as a reference. The mean ± SD in the graph shows the relative levels from three replicates. **p < 0.05, **p < 0.01.*
